# Essential tremor patients experience significant burden beyond tremor: A systematic literature review

**DOI:** 10.3389/fneur.2022.891446

**Published:** 2022-07-22

**Authors:** Margaret E. Gerbasi, Shruti Nambiar, Spencer Reed, Kalin Hennegan, Nandini Hadker, Adi Eldar-Lissai, Stephanie Cosentino

**Affiliations:** ^1^Sage Therapeutics, Inc., Cambridge, MA, United States; ^2^Trinity Life Sciences, Waltham, MA, United States; ^3^Cognitive Neuroscience Division of the Department of Neurology, Gertrude H. Sergievsky Center, Taub Institute for Research on Alzheimer's Disease and the Aging Brain, Columbia University Medical Center, New York, NY, United States

**Keywords:** essential tremor, burden of illness, systematic literature review, non-motor symptoms, quality of life

## Abstract

**Background:**

Essential tremor (ET) is among the most common movement disorders in adults. While ET is diagnosed and primarily characterized by the presence of tremor, it also can impact cognition, sleep, mood, and motor functioning more broadly. The manifestations of ET can have various consequences, including difficulty with activities of daily living (ADL), embarrassment, and overall decline in health-related quality of life, which have not been fully explored in prior studies.

**Objective:**

We performed a systematic literature review to comprehensively characterize the burden experienced by patients with ET from the clinical and humanistic perspectives, focusing on outcomes beyond tremor.

**Methods:**

This systematic literature review followed Preferred Reporting Items for Systematic Reviews and Meta-Analyses (PRISMA) guidelines. Searches in PubMed, Embase, and Cochrane Library identified original, observational studies of the clinical and humanistic burden in adult patients with ET published in English between 2010 and 2020. Studies assessing epidemiology, treatment patterns, or disease management were excluded. Search results were screened according to pre-determined eligibility criteria. Data from included studies were collected, independently verified, and qualitatively synthesized.

**Results:**

Following the screening of 2,303 records and 145 full-text articles, 39 studies were identified. There was significant heterogeneity in study designs, statistical approaches, and patient cohorts across the included studies. Patients with ET in these studies exhibited more severe disabilities and reduced independence compared to healthy individuals, and they often struggled to perform ADL and relied on caregivers for physical and emotional support. Patients also experienced various issues with movement and balance, increased risk of falls, depression, anxiety, poor sleep quality, and psychosocial consequences including embarrassment, apathy, and enfeeblement.

**Conclusion:**

A systematic literature review of non-tremor manifestations and/or consequences of ET identified far-reaching negative impacts on patients' ability to function independently and revealed accompanying psychosocial effects, including social fear and embarrassment. The reduced function and psychosocial deficits observed in patients with ET result in significant clinical and humanistic burdens, decreasing quality of life. Future studies should evaluate this condition beyond the tremor itself to provide an improved understanding of the multi-dimensional burden of the disease, thereby highlighting the need to diagnose and appropriately manage patients with ET.

## Introduction

Essential tremor (ET) is among the most common movement disorders, with an estimated global prevalence of about 13.3 per 1,000 persons, increasing to 57.9 per 1,000 persons for those 65 years of age and older (1, 2). In the United States (US) alone, an estimated 6.4 million adults are affected by ET, although the reported prevalence varies significantly, potentially due to variable presentation as well as differences in diagnostic criteria (3–6). ET has a bimodal age of onset, with some patients developing ET in early adulthood and others later in life (7). ET is characterized by kinetic and postural tremor, predominantly in the upper limbs (8). However, patients with ET may also exhibit tremor of the head, voice, trunk, and lower limbs (9, 10), as well as other non-tremor motor symptoms like gait difficulties (11), non-motor symptoms, including cognitive difficulties (12), psychiatric symptoms (13), sensory impairment (14), and sleep disturbances (13). Together, these symptoms contribute to the overall burden of disease, suggesting that ET could be defined holistically as a multi-system disease that extends beyond just tremor.

Propranolol, the only pharmacological therapy approved by the US Food and Drug Administration (FDA) for limb tremor associated with ET, obtained approval in 1967; however, the majority of patients do not adequately respond to propranolol leaving them with substantial uncontrolled tremor, highlighting the existing unmet need for ET therapies (10, 15). Off-label therapies, such as primidone, topiramate, alprazolam, and botulinum toxin Type A injections have been used based on limited evidence (16). FDA-approved procedures, such as deep brain stimulation and MRI guided focused ultrasound, offer alternative options for medication-refractory patients (16), but their long-term efficacy remains unclear (17). Notably, the efficacy of existing ET therapies has been defined by their ability to reduce the frequency or severity of upper limb tremor only, and there is a lack of treatment options that can address other known clinical and humanistic manifestations and/or consequences of the disease (16).

The combination of tremor and non-tremor manifestations of ET may cause patients to experience social isolation or embarrassment and may result in avoidance of social interactions and certain activities in public, such as eating, drinking, or writing (18). Existing generic instruments that measure ADL may not completely capture specific limitations patients experience in performing their daily tasks due to ET (19, 20). The inability to perform tasks impacted directly by upper limb tremors, such as tasks requiring hand manipulation of objects as well as those for communication, cognitive processes, or profession, is crucial to capture and could be attained using ET-specific ADL measures (21). Although patients with ET experience a heterogenous set of manifestations that can combine to impact their health-related quality of life (HRQOL), prior research on the burden of ET has focused on tremor or individual specific non-tremor symptoms (e.g., cognition, mood, non-tremor motor function, sleep) in isolation. The objective of this systematic literature review was to look beyond tremor and comprehensively examine the burden of ET from a clinical and humanistic perspective.

## Methods

A comprehensive systematic literature review was performed in accordance with the Preferred Reporting Items for Systematic Reviews and Meta-Analyses (PRISMA) guidelines on reporting standards for qualitative and quantitative reviews (22).

### Data sources

Systematic searches were run across PubMed (*via* pubmed.com), Embase (*via* embase.com), and CENTRAL and CDSR (*via* Cochrane Library) to identify peer-reviewed literature published from January 2010 through December 2020. Search algorithms were designed for each database using appropriate syntax, with a combination of medical subject headings and free text in titles and abstracts of records (see search strings in Supplementary Table 1). Conference proceedings from 2017 to 2020 were also included in the search, but inclusion in the review was dependent on the availability of adequate data for extraction. The review was limited to English-language papers, but the geographical region of the studies was not restricted.

### Study selection

Records identified from each database were pooled, and duplicate records were removed using EndNote version X9.3.3. Records were reviewed for inclusion based on defined eligibility criteria (see Supplementary Table 2). Studies for inclusion had to be non-interventional in nature and conducted in adult patients with ET. The use of any structured questionnaire that helps in the quantification of the clinical or humanistic manifestations and/or consequences of ET and the availability of data on the scores of such a questionnaire were key requirements for inclusion of studies in this review. Validation or psychometric analysis of new instruments or studies testing the level of correlation between different instruments were excluded. Studies with sample size of less than 30 ET patients were excluded due to concerns of generalizability.

Record screening was conducted in two phases: title/abstract screening and full-text screening. Title/abstract screening was conducted by a single researcher, with 15% of excluded abstracts reviewed by a second researcher for quality control using a hierarchical approach for assigning reasons for exclusion. Full-text articles were independently screened by two researchers per the eligibility criteria. Disagreements between the researchers on inclusion of records were resolved *via* discussion, third-party resolution, or by a senior researcher.

### Data extraction and analysis

Data from included papers were extracted by one researcher, and all data points were independently validated by another researcher. Data that specifically answered the research questions of interest were extracted, including publication details, study characteristics, patient characteristics, and outcomes of interest. Data extracted from studies were grouped into key outcome categories that can contribute to the patient burden of ET: cognitive impairment, psychosocial manifestations and consequences, sleep disturbance and fatigue, non-tremor motor dysfunction causing gait and balance issues and falls, impact on ADL, and impact on HRQOL.

The identified literature was synthesized qualitatively to discuss the comprehensive burden experienced by patients with ET. No quantitative analysis was conducted due to variations in outcomes measured across studies. Statistical significance reported in this review was based on the criteria of the individual included studies. Risk of bias assessment was not performed due to the differences in the objectives, study design, and outcomes of the included studies.

## Results

### Search results

**Figure 1:**
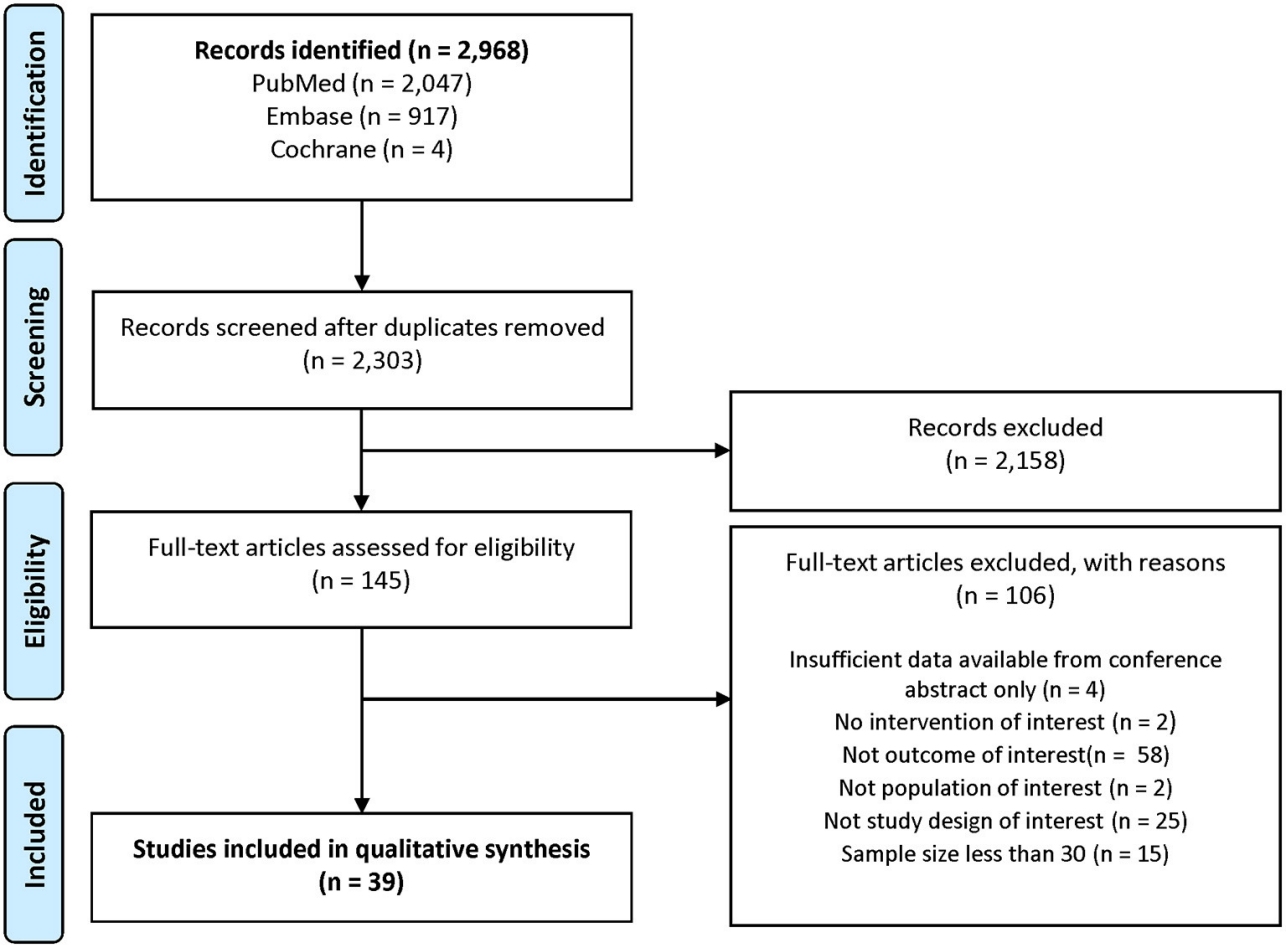
PRISMA study attrition diagram.

The database searches identified 2,303 records eligible for screening after deduplication. Title/abstract screening resulted in 145 articles for full-text review, 39 of which were included for the evidence synthesis in this review (Figure 1).

The included studies varied in populations, study designs, and specific questionnaires for measurement of outcomes. More than half of the studies (54%) were cross-sectional surveys, and 18% of the studies were described as prospective, longitudinal, observational studies. The remaining studies were epidemiological studies with no information on follow-up periods. Of the 39 studies, 16 studies recruited cohorts in a case-control design. Additional study and patient characteristics are provided in Supplementary Table 3.

The mean age of the ET cohorts in the included studies ranged from 25 years (23) to 87 years (24). Nine studies reported data from the Clinical Pathological Study of Cognitive Impairment in Essential Tremor (COGNET) study, an ongoing study in the US (24–32). These studies covered different enrollment periods, research questions, and/or subpopulations of the larger study cohort, but there is potential overlap in patients across these studies.

**Figure 2:**
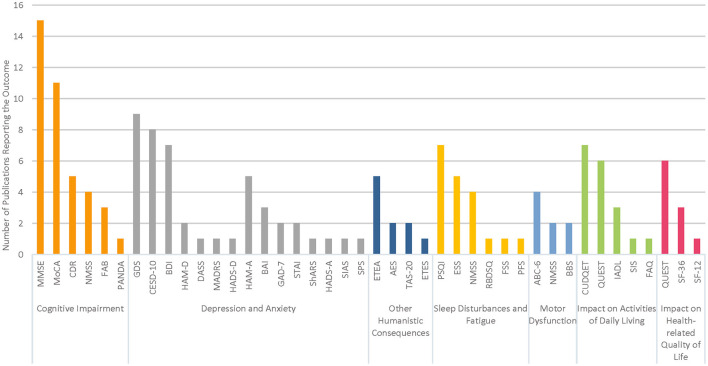
Summary of structured instruments reported for each outcome category.

Details of the structured questionnaires used to assess each outcome category are provided in Table 1. The number of publications reporting data using these instruments is illustrated in Figure 2 for each outcome category of interest. Cognitive impairment and depression were the most frequently examined outcomes across the 39 studies.

**Table 1:** Description of structured instruments reported in included studies.

**Outcome category**	**Structured** **instrument**	**Instrument** **abbreviation**	**Instrument description complied from included studies**	**Studies** **reporting**
Cognitive impairment	Folstein Mini Mental State Examination	MMSE	30-point, 11-item clinician-administered assessment of orientation, attention, short term memory recall, language and visuoconstruction. Lower scores indicate more severe cognitive impairment (modified versions also reported)	(11, 24, 26, 33–44)
	Montreal Cognitive Assessment	MoCA	30-point, 30-item clinician-administered assessment of executive function, visuoconstruction, language, memory, attention, verbal fluency, and abstraction. Lower scores indicate more severe cognitive impairment	(23, 25–29, 31, 32, 42, 44, 45)
	Clinical Dementia Rating	CDR	3-point, 6-domain (Sum of Boxes scores range from 0 to 18 but often reported as a global score between 0 and 3) clinician-administered scale assessing memory, orientation, judgment and problem solving, community affairs, home and hobbies, and personal care from the perspective of the patient and caregiver. Higher scores indicate more severe cognitive impairment	(25, 26, 28, 29, 31, 32)
	Non-Motor Symptoms Scale-Attention/Memory Domain	NMSS	360-point, 30-item clinician-rated scale encompassing 9 domains related to non-motor symptoms of movement disorders experienced in the past month; the score for each domain is based on multiple levels of severity from 0 to 3 and frequency scores from 1 to 4; the attention/memory domain was used to assess cognitive impairment. Higher scores indicate more impairment	(42, 45–47)
	Frontal Assessment Battery	FAB	18-point, 6-item clinician-administered assessment of conceptualization, mental flexibility, motor programming, sensitivity to interference, and inhibitory control. Lower scores indicate greater dysfunction	(35, 44, 48)
	Parkinson Neuropsychometric Dementia Assessment	PANDA	Clinician reported assessment for the detection of cognitive deficits designed for Parkinson's Disease, with evaluation of verbal fluency, word pair association learning with immediate and delayed recall, visuospatial perception, and working memory testing	(48)
Depression and anxiety	Geriatric Depression Scale	GDS	30-point, 30-item patient-reported measure of depressive symptoms. Higher scores indicate more severe depression	
	10-item Center for Epidemiological Studies Depression Scale	CESD-10	30 point, 10-item patient-reported assessment for evaluating depression. Higher scores indicate more severe depression	(11, 30, 31, 34, 35, 49–51)
	Beck Depression Inventory	BDI	63-point, 21-item patient-reported assessment to measure depressive symptoms within the last week. Higher scores indicate more severe depression	(23, 36, 45, 48, 52–54)
	Hamilton Depression Rating Scale	HAM-D	52-point, 21-item clinician-reported assessment of depressive symptoms, scoring only 17 items. Higher scores indicate more severe depression	(41, 55)
	Depression Anxiety Stress Scale	DASS	126-point, 42-item patient-reported scale to measure depression, anxiety, and stress/tension. Higher scores indicate greater severity	(53)
	Montgomery-Asberg Depression Rating Scale	MADRS	60-point, 10-item clinician-reported assessment evaluating the core symptoms of depression experienced over the past week. Higher scores indicate more severe depression	(42)
	Hospital Anxiety and Depression Scale - Depression	HADS-D	42-point, 14-item patient-reported scale to assess symptoms of anxiety and depression (7 questions each). Higher scores indicate more severe symptoms	(44)
	Hamilton Anxiety Rating Scale	HAM-A	56-point, 14-item clinician-reported scale to assess the level, distribution, and change in patient's anxiety symptoms. Higher scores indicate greater anxiety (modified versions also reported)	(41, 45, 55–57)
	Beck Anxiety Inventory	BAI	63-point, 21-item patient-reported assessment to evaluate anxiety based on symptom severity. Higher scores indicate greater anxiety	(23, 42, 54)
	Generalized Anxiety Scale	GAD-7	21-point, 7-item patient-reported scale for assessing the severity of various anxiety symptoms. Higher scores indicate greater anxiety	(25, 26)
	State-Trait Anxiety Inventory	STAI	80-point, 20-item patient-reported assessment to help differentiate anxiety as a condition from anxiety as a personality trait. Higher scores indicate greater anxiety	(48, 53)
	Sheehan Clinical Anxiety Rating Scale	ShARS	140-point, 35-item patient- or clinician-reported assessment of 16 core anxiety symptoms and 19 symptoms and behaviors associated with range of panic disorder clinical presentations. Also known as the Sheehan Panic Disorder Scale	(44)
	Hospital Anxiety and Depression Scale - Anxiety	HADS-A	42-point, 14-item patient-reported scale to assess symptoms of anxiety and depression (7 questions each). Higher scores indicate more severe symptoms	(44)
	Social Interaction Anxiety Scale	SIAS	80-point, 20-item patient-reported assessment to evaluate distress when meeting and conversing with others. Higher scores indicate greater anxiety	(53)
	Social Phobia Scale	SPS	80-point, 20-item patient-reported scale to assess fear of scrutiny during daily, routine activities. Higher scores indicate greater anxiety	(53)
Other humanistic consequences	Essential Tremor Embarrassment Assessment	ETEA	70-point, 14-item self-reported assessment of tremor related embarrassment. Higher scores indicate greater feelings of embarrassment	(25, 28, 49–51)
	Apathy Evaluation Scale	AES	72-point, 18-item patient-reported scale to assess feeling of apathy over the past 4 weeks. Higher scores indicate more severe apathy	(35, 48)
	Toronto Alexithymia Scale	TAS-20	100-point, 20-item patient-reported scale to assess difficulty recognizing and expressing emotions. Higher scores indicate greater alexithymia	
	Essential Tremor Enfeeblement Scale	ETES	40-point, 8-item caregiver-rated enfeeblement in ET patients. Higher scores indicate more enfeeblement	(25)
Sleep disturbances and fatigue	Pittsburgh Sleep Quality Index	PSQI	21-point, 19-item patient-reported (plus 5 additional questions for the bed-partner-/roommate if available) scale to assess sleep quality and disorder over the past month. Higher scores indicate more severely disturbed sleep	(23, 24, 42, 45, 49, 55, 57)
	Epworth Sleepiness Scale	ESS	24-point, 8-item patient-reported scale on likelihood a patient may fall asleep in common situations. Higher scores indicate greater daytime sleepiness	(23, 24, 42, 48, 55)
	Non-Motor Symptoms Scale-Sleep/Fatigue Domain	NMSS	360-point, 30-item clinician-rated scale encompassing nine domains related to non-motor symptoms of movement disorders experienced in the past month; the score for each domain is based on multiple levels of severity from 0 to 3 and frequency scores from 1 to 4; the sleep/fatigue domain is used to assess sleep disturbances and fatigue. Higher scores indicate more impairment	(42, 45–47)
	REM Sleep Behavior Disorder Screening Questionnaire	RBDSQ	13-point, 10-item patient-reported instrument that assesses the subject's sleep behavior related to the frequency and contents of dreams and their relationship to movements during sleep, self-injuries and injuries to the bed partner, motor behavior while asleep (talking, sudden movements), awakening and disturbed sleep and finally, the presence of any neurological disorder. Higher scores indicate more disturbed sleep	(58)
	Fatigue Severity Scale	FSS	63-point, 9-item patient-reported scale assessing fatigue severity and its disruption on certain activities. Higher scores indicate greater fatigue	(23)
	Parkinson's Disease Fatigue Scale	PFS	16-point, 16-item patient-reported scale assessing the physical effects of fatigue and their impact on daily functioning. Higher scores indicate greater fatigue	(55)
Motor dysfunction: gait, balance and falls	Activities-specific Balance Confidence Scale	ABC-6	100-point, 6-item patient-reported scale assessing patient's confidence in performing six activities without losing their balance. Lower scores indicate worse balance confidence	(11, 29, 32, 38)
	Non-Motor Symptoms Scale	NMSS	360-point, 30-item clinician-rated scale encompassing nine domains related to non-motor symptoms of movement disorders experienced in the past month; the score for each domain is based on multiple levels of severity from 0 to 3 and frequency scores from 1 to 4; the item “Falls due to fainting” in the cardiovascular domain was used to assess falls. Higher scores indicate more impairment	(46, 47)
	Berg Balance Scale	BBS	56-point, 14-item clinician-reported assessment of a patient's ability to maintain balance while performing a variety of tasks such as standing on one foot or picking up an object from the ground. Lower scores indicate worse balance	(11, 44)
Impact on activities of daily living	Columbia University Disability Questionnaire for Essential Tremor	CUDQET	100-point, 36-item patient-reported scale assessing patient disability completing a range of activities of daily living such as carrying a cup, tying shoelaces, signing name, etc. Higher scores indicate greater disability (modified versions also reported)	(25, 26, 28, 30, 31, 49, 59)
	Quality of Life in Essential Tremor Questionnaire	QUEST	100-point, 30-item patient-reported assessment of quality of life across five domains: physical, psychosocial, communication, hobbies/leisure, and work/finance. All domains except the psychosocial domain were used to assess activities of daily living	(34, 47, 49, 51, 55, 56)
	Lawton Instrumental Activities of Daily Living Scale	IADL	8-point, 8-item patient-reported assessment of patient independence performing tasks such as cooking, housekeeping, finances, laundry, medication management, etc. Lower scores indicate a higher level of dependence	(25–27)
	Subjective Incompetence Scale	SIS	36-point, 12-item patient-reported scale assessing the frequency and severity of incompetence during the last week. Higher scores indicate greater feelings of incompetence	(26)
	Pfeffer Functional Activities Questionnaire	FAQ	30-point, 10-item caregiver-rated assessment of 10 common activities that require complex cognitive and social functioning. Higher scores indicate a higher level of dependence	(40)
Impact on health-related quality of life	Quality of Life in Essential Tremor Questionnaire	QUEST	100-point, 30-item patient-reported assessment of quality of life across five domains: physical, psychosocial, communication, hobbies/leisure, and work/finance	(34, 47, 49, 51, 55, 56)
	36-item Short Form Survey	SF-36	100-point, 36-item patient-reported assessment of physical functioning, physical and emotional limitations, social functioning, bodily pain, and general and mental health. Higher scores indicate more favorable health status	(23, 45, 48)
	12-item Short Form Survey	SF-12	100-point, 12-item patient-reported modified version of the SF-36. Higher scores indicate more favorable health status	(52)

### Outcomes reported in studies

#### Cognitive impairment

Cognitive impairment was reported in a total of 26 studies across the US (11, 24–29, 31–38), Europe (23, 39, 40, 48), Asia (41–44, 46, 47), and Africa (45). The mean patient ages ranged from 25 to 87 years across included studies; eight publications assessed cognitive impairment specifically in older populations with mean patient ages >80 years (24, 25, 29, 32, 33, 36–38). Cognitive impairment was measured using a variety of scales, including versions of the Folstein Mini Mental State Examination (MMSE), including the MMSE, MMSE-37, and modified MMSE (11, 24, 26, 33–44); the Montreal Cognitive Assessment (MoCA) (23, 25–29, 31, 32, 42, 44, 45); the Clinical Dementia Rating Scale (CDR) (25, 26, 29, 31, 32); the Frontal Assessment Battery (FAB) (35, 44, 48); the Parkinson Neuropsychometric Dementia Assessment (PANDA) (48); and the attention/memory domain of the Non-Motor Symptoms Scale (NMSS) (42, 45–47). Modified versions of these instruments were used to accommodate low education levels or to differentiate cognitive functioning across varying stages of dementia (36, 38–40).

Overall, patients with ET demonstrated and reported lower cognitive performance compared to controls across the studies, although the differences were not always statistically significant (23, 39, 40, 42, 45, 47, 48). Mean MoCA scores lower than 24 indicate the presence of cognitive impairment, and one study reported 42.5% patients had such impairment (27). Studies also reported that ET patients performed lower on certain sub-scores from the MMSE and MoCA including attention/calculation, construction, orientation, language, recall, visuospatial/executive, and abstraction (23, 38, 42). In a cross-sectional study from China, 42% of patients with ET reported experiencing impaired concentration, while 60 and 27% of patients reported difficulties remembering things or events and remembering to do things, respectively (46). Patients with ET with midline tremor reported experiencing more impairment related to concentrating, remembering to do things, and remembering things or events compared to patients without midline tremor as assessed by the attention/memory domain of the NMSS (46). Similarly, patients with ET with head tremor reported more difficulty remembering to do things compared to ET patients without head tremor (47). With regard to objective test performance, patients with ET demonstrated a variety of cognitive impairments. Examination of MoCA subscores, for example, revealed weaker performance on the language, executive functioning, abstract, and delayed recall domains compared to controls.

Caregiver-rated scores using the CDR found that though the majority of patients in study cohorts (62–80%) had normal cognition (25, 29, 31, 32), 15–29% had mild cognitive impairment (25, 26, 29, 31, 32), and 5–11% patients had CDR score ≥1, indicating at least mild dementia (25, 29, 31, 32). Cognitive impairment has been associated with features of ET including later age of disease onset and greater tremor severity (36). Moreover, certain tremor features have been associated with subjective reports of impaired cognition in ET. Specifically, ET patients with midline tremor reported experiencing more impairment related to concentration and memory, including remembering tasks, things, or events, compared to patients without midline tremor, as assessed by the attention/memory domain of the NMSS (46). Patients with head tremor reported having more difficulty in remembering tasks compared to those without head tremor (47).

#### Psychosocial manifestations and consequences

##### Depression and anxiety

Depression and anxiety are among the most common non-motor symptoms of ET and can add to the emotional and mental burden of patients. Depression in patients with ET was assessed in 28 publications using a variety of outcome measures. Most of the studies examining depression were conducted in the US (11, 24–32, 34–36, 49–51), but there were five studies from Europe (23, 48, 52–54), six from Asia (41, 42, 44, 55, 56), and one each from Africa (45) and Canada (58). Mean patient age across 21 studies in overall ET cohorts ranged from 40.7 to 79.0 years (11, 26–28, 30, 31, 34, 35, 41, 42, 44, 48–56, 58). The remaining seven publications assessed depression in subgroups of patients with ET stratified by age group or cognitive status (23–25, 29, 32, 36, 45). Patients with ET were assessed using the Geriatric Depression Scale (GDS), 10-item Center for Epidemiological Studies Depression Scale (CESD-10) (11, 30, 31, 34, 35, 49–51), Beck Depression Inventory (BDI) (23, 36, 45, 48, 52–54), Hamilton Depression Rating Scale (HAM-D) (41, 55), Depression Anxiety Stress Scale (DASS) (53), Montgomery-Åsberg Depression Rating Scale (MADRS) (42), and Hospital Anxiety and Depression Scale – Depression (HADS-D) (44).

In most studies, patients with ET exhibited higher statistically significant mean scores on validated depression instruments compared to controls, indicating higher levels of depression (35, 42, 45, 48, 53–55). The proportion of patients who experienced depression is reported by level of severity in Table 2. Between 52 and 60% of patients with ET had mild, moderate, or severe depression compared to 17−37% of controls as assessed by the BDI in two studies (23, 54). Across four independent studies, approximately one in five patients scored in the significantly depressed range (the highest rating on the GDS scale), ranging from 19 to 22% patients (25, 26, 41, 58). In a study using age-matched controls, patients with ET had statistically significantly greater depressive symptoms (45). Depression has been reported to be statistically associated with tremor disability and with neck, face, voice, and cranial tremor (41). Other work, however, has suggested that depressive symptoms are dissociated from tremor severity, clustering with cognitive and ADL impairment instead (27).

**Table 2:** Prevalence of varying severity of depression and anxiety.

**Publication**	**Country**	**Population**	**Sample size**	**Outcome measure**	**Mild (%)**	**Moderate (%)**	**Severe (%)**
Chandran et al. (55)	India	ET patients	50	HAM-D^*^	32.0%	10.0%	2.0%
		Controls	50		6.0%	2.0%	0.0%
Huang et al. (41)	China	ET Patients	245	HAM-D^**^	40.0%	14.3%	
Louis et al. (50)	US	ET Patients	91	CESD-10^†^	–	40.7%	7.7%
Louis et al. (34)	US	ET patients	70	CESD-10^†^	–	34.3%	7.1%
Sengul et al. (23)	Turkey	ET patients	45	BDI^δ^	24.4%	22.2%	13.3%
		Controls	35		11.4%	2.9%	2.9%
Sengul et al. (54)	Turkey	ET patients	100	BDI^δ^	31.6%	12.2%	8.2%
		Controls	100		23.0%	8.0%	6.0%
Acar and Acar (57)	Turkey	ET patients	40	HAM-A^α^	40.0%	50.0%	0.0%
		Controls	38		36.8%	0.0%	0.0%
Chandran et al. (55)	India	ET patients	50	HAM-A^‡^	52.0%	8.0%	6.0%
		Controls	50		18.0%	0.0%	0.0%
Huang et al. (41)	China	ET Patients	245	HAM-A ^γ^	38.0%	25.3%	
Sengul et al. (23)	Turkey	ET patients	45	BAI ^ζ^	20.0%	28.9%	42.2%
		Controls	35		20.0%	20.0%	0.0%
Sengul et al. (54)	Turkey	ET patients	100	BAI ^ε^	24.5%	20.2%	26.6%
		Controls	100		18.0%	12.0%	9.0%

*BAI, Beck Anxiety Inventory; BDI, Beck Depression Inventory; CESD-10, 10-item Center for Epidemiological Studies Depression Scales; ET, Essential tremor; HAM-A, Hamilton Anxiety Rating Scale; HAM-D, Hamilton Depression Rating Scale; SD, Standard deviation; US, United States.*

* *Cut-offs for mild, moderate, and severe depression were as follows: mild = 7–17; moderate = 18–24; severe = >24.*

** *Cut-offs for mild and moderate-severe depression were as follows: mild = 8–20; moderate to severe = >20.*

† *Cut-offs for moderate and severe depression were as follows: moderate = 10–19; severe ≥ 20.*

δ *Cut-offs for mild, moderate, and severe depression were as follows: mild = 14–19; moderate = 20–28; severe = 29–63.*

α *Cut-offs for mild, moderate, and severe anxiety were as follows: mild = 6–14; moderate = 15–24; severe = >24.*

‡ *Cut-offs for mild, moderate, and severe anxiety were as follows: mild ≤ 17; moderate = 18–24; severe = 25–30.*

γ *Cut-offs for mild and moderate-severe anxiety were as follows: mild = 7–14; moderate to severe = >14.*

ζ *Cut-offs for mild, moderate, and severe anxiety were as follows: mild = 8–15; moderate = 16–25; severe = 26–63.*

ε *Cut-offs for mild, moderate, and severe anxiety were as follows: mild = 10–16; moderate = 17–29; severe = 30–63*.

Anxiety was assessed in 13 publications from the US (25, 26), Europe (23, 48, 53, 54, 57), and Asia (41, 42, 44, 55, 56), and Africa (45). Across these publications, the average patient age ranged from 25–81 years (23, 25, 26, 41, 42, 44, 45, 48, 53–57). Anxiety was assessed using a total of eight scales, including versions of the Hamilton Anxiety Rating Scale (HAM-A) (41, 45, 55–57), Beck Anxiety Inventory (BAI) (23, 42, 54), Geriatric Anxiety Disorder 7-item scale (GAD-7) (25, 26), State-Trait Anxiety Inventory (STAI) (48, 53), Hospital Anxiety and Depression Scale – Anxiety (HADS-A) (44), Sheehan Clinical Anxiety Rating Scale (ShARS) (44), Social Interaction Anxiety Scale (SIAS) (53), and Social Phobia Scale (SPS) (53). In some studies, depression and anxiety were reported in conjunction with psychosocial issues like embarrassment, demoralization and enfeeblement, which were correlated (25, 26, 28, 50).

Overall, patients with ET generally experienced greater statistically significant anxiety based on mean scores across BAI, DASS, and HAM-A compared to healthy controls (23, 42, 45, 48, 53–55, 57). Symptoms of anxiety were more severe in patients with neck, face, voice, or cranial tremor (41). A larger proportion of patients with ET demonstrated at least mild anxiety (52%) compared with controls (18%) (55). A higher proportion of patients with ET also had moderate-to-severe anxiety compared to controls, although statistical significance was not tested (23, 54, 55, 57). The proportion of patients experiencing anxiety by degree of severity varied across studies due to the differences in study designs and sample characteristics; however, the presence of anxiety, even mild, as a symptom among patients with ET is noteworthy.

##### Other humanistic consequences

Essential tremor can also impact other humanistic aspects of psychosocial well-being that may be overlooked in the context of clinical manifestations like depression and anxiety. Feelings of embarrassment, alexithymia (the inability to experience feelings), apathy, and enfeeblement (i.e., premature feelings of helplessness) were assessed in 13 publications across the US (25, 26, 28, 35, 49–51), Europe (48, 54), Asia (42, 46, 47), and Africa (45), but limited data were available on each outcome. The mean patient age across publications ranged from 44 to 81years (25, 26, 28, 35, 42, 45–51, 54). Included studies addressed the following psychosocial outcomes: embarrassment associated with ET (assessed by the Essential Tremor Embarrassment Assessment; ETEA) (25, 28, 49–51), apathy (assessed by the Apathy Evaluation Scale; AES) (35, 48), alexithymia (assessed by the Toronto Alexithymia Scale; TAS-20), and enfeeblement (assessed by the Essential Tremor Enfeeblement Scale; ETES) (25).

The limited data on these additional psychosocial outcomes indicated that patients with ET experienced negative effects on their emotional and social well-being. Two US studies reported embarrassment using the ETEA scale, and notably, self-reported scores from patients with ET indicated higher levels of embarrassment than caregiver scores provided using the caregiver version of the ETEA scale (25, 28). Other studies conducted only in patients with ET also reported that patients experienced embarrassment that increased if they also had depressive symptoms (49–51). Greater embarrassment in patients with ET was also shown to be associated with higher tremor disability score (28).

Patients with ET experienced greater overall feelings of apathy compared to controls as assessed by the AES (35, 48). Compared to controls, higher TAS-20 total as well as domain scores were reported for patients with ET. Half the patients with ET had definite or probable alexithymia measured by TAS-20 compared to 30% of controls. Higher ETES scores, showing caregiver-rated enfeeblement, were positively associated with tremor severity and disability, functional and gait disability, greater cognitive difficulty, and increased depressive symptoms highlighting how a patient's dependence on a caregiver may contribute to the caregiver's burden (25). Although these findings are from a limited number of studies, they provide directional evidence that patients with ET can experience psychosocial issues apart from depression and anxiety that add to the overall clinical and humanistic burden of the disease.

#### Sleep disturbances and fatigue

Sleep disturbances and fatigue were assessed in 11 publications across the US (24, 49), Europe (23, 48, 57), Asia (42, 46, 47, 55), Africa (45) and Canada (58). The average patient age ranged from 25–87 years (23, 24, 42, 45–49, 55, 57, 58). Various structured questionnaires were utilized to assess the effect of ET on sleep and fatigue, including the Pittsburgh Sleep Quality Index (PSQI) (23, 24, 42, 45, 49, 55, 57), Epworth Sleepiness Scale (ESS) (23, 24, 42, 48, 55), the sleep/fatigue domain of the NMSS (42, 45–47), the REM Sleep Behavior Disorder Screening Questionnaire (RBDSQ) (58), the Fatigue Severity Scale (FSS), and the Parkinson's Disease Fatigue Scale (PFS) (55). The prevalence and severity of disturbed sleep and fatigue were similar across all geographies.

Overall, patients with ET experienced significantly greater fatigue and disturbed sleep compared to healthy controls. PSQI scores ranged from 5.9 to 6.83 for patients with ET and from 2.6 to 5.41 for controls (23, 42, 45, 55, 57). Patients with midline or head tremor showed overall greater severity and prevalence of sleep disturbances and fatigue compared to patients without midline or head tremors, particularly related to difficulty falling asleep (46, 47). Patients with midline tremor (48–56%) also experienced more daytime sleepiness compared to patients without midline tremor (36%) (46).

#### Motor dysfunction: Gait, balance and falls

Patients with ET experience other motor symptoms beyond tremor, such as gait issues, trouble maintaining balance, and a propensity for falls. These movement-related outcomes were assessed in 12 publications across the US (11, 25, 29, 31, 32, 37, 38), Europe (60), and Asia (43, 44, 46, 47). The average patient age ranged from 44 to 86 years (11, 25, 29, 31, 32, 37, 38, 43, 44, 46, 47, 60). Clinician-reported, performance-based metrics, such as the tandem index, number of missteps, and tandem walk test were used to assess gait issues. Balance was reported in five publications, using the patient-reported Activities-specific Balance Confidence Scale – 6-item version (ABC-6) (11, 29, 32, 38) or the performance-based Berg Balance Scale (BBS) (11, 44), with one study reporting both scales. Propensity for falls was assessed using either the Non-Motor Symptoms Scale (NMSS) (46, 47) or the absolute number of falls (11, 29, 31, 32, 38).

Tandem missteps were defined as the number of steps that fall out of a straight line when participants walk placing one foot in front of the other, touching heel to toe in a straight line (32). Patients with ET had greater statistically significant number of tandem missteps when compared to controls (37, 60). Gait was also assessed by the mean (SD) number of missteps during a 10-step, or 3-meter standard and tandem walk tests. The scores ranged from 4.9 to 5.7 for patients with ET in a 10-step tandem walk test (25, 32), and 4.4 (4.7) vs. 2.2 (3.7) for patients with ET vs. controls during a 3-meter tandem walk test (37), respectively. Patients with ET were able to take fewer steps before a misstep, with a mean number of steps (SD) of 8.5 (4.5) vs. 10.6 (3.9) for patients with ET vs. controls in a 15-step tandem walk test (60). Additionally, patients with ET had slower gait velocity during standard and tandem walk tests and took statistically significantly fewer steps per minute in both walk tests compared to controls (37). The number of tandem missteps was significantly correlated with total tremor score measured by the Fahn–Tolosa–Marin Tremor Rating Scale (FTMTRS) (60).

Overall, balance confidence was statistically significantly lower in patients with ET compared to controls in both studies examining this measure *via* either the ABC-6 or BBS (11, 38). Balance confidence was lower for patients with ET who had head tremor and low cognitive performance (11, 38) compared to controls and patients without these characteristics. Lower balance confidence, while positively correlated with the presence of head tremor, was not correlated with age of tremor onset or duration of disease (11). Patients with head tremor reported low balance confidence vs. controls when walking on icy sidewalks (66.7 vs. 39.2%), standing on a chair and reaching for something (53.3 vs. 23.2%), and stepping on or off an escalator without holding the rail (46.7 vs. 24.4%) (11).

The average number of falls in the past year ranged from 0.6 to 2.2 for patients with ET compared to 0.59–0.6 for controls across studies (11, 29, 31, 32, 38). Patients with low cognitive performance had a statistically significantly greater mean number of falls per year compared to patients with high cognitive performance and controls (11, 29, 31, 32, 38). A higher proportion of patients with low cognitive performance also had a fall in the last 12 months compared to the two other cohorts (38).

#### Impact on ADL

ET has a substantial impact on patients' abilities to perform day-to-day activities and function independently. A total of 14 publications across the US (25–28, 30, 31, 34, 49, 51, 59), Europe (40), and Asia (47, 55, 56) assessed the impact of ET on ADL. The average patient ages across publications ranged from 41 to 81 years (25–28, 30, 31, 34, 40, 49, 51, 59). ADL was assessed using a variety of both disease agnostic and ET-specific questionnaires and scales, including the Columbia University Disability Questionnaire for Essential Tremor (CUDQET) (25, 26, 28, 30, 31, 49, 59), subscales of Quality of Life in Essential Tremor Questionnaire (QUEST) (34, 47, 49, 51, 55, 56), the Lawton Instrumental Activities of Daily Living Scale (IADL) (25–27), the Subjective Incompetence Scale (SIS) (26), and the Pfeffer Functional Activities Questionnaire (FAQ) (40). Assessments using QUEST captured ADL through the physical, communication, work/finance, psychosocial, and hobbies/leisure domains.

Three publications that assessed patients from the COGNET study, with average ages greater than 65 years, reported mean Lawton IADL scores >7, indicating high levels of independence in performing daily activities like cooking, housekeeping, shopping, transportation, finances, laundry, managing medication, and using the telephone (25–27). Greater disability due to tremor was significantly correlated with more waking hours experiencing tremor as well as age of onset of tremor (28, 49, 59).

Questionnaires focused more specifically on fine motor activities, or the extent to which ET interferes with such activities, reveal higher levels of functional impairment. On the full version of the CUDQET, mean values for patients with ET ranged from 53.6 to 67.6 (out of a possible 100 points) in four publications, indicating substantial impairment in the self-reported ability of patients to perform a variety of motor activities, such as writing out a signature, carrying a cup, etc. (Table 3) (25, 26, 28, 49). Patients who had experienced tremors for ≥40 years had significantly greater impairment than those who had experienced tremors for 0–9 years (59).

**Table 3:** Mean Columbia University Disability Questionnaire for Essential Tremor scores.

**Publication**	**Country**	**Population**	**Sample size**	**Mean (SD)**
Cersonsky et al.^†^ (25)	US	ET patients	98	65.1 (24.8)
Cersonsky et al.^†^ (26)	US	ET patients	60	64.2 (24.9)
Kellner et al.^†^ (28)	US	ET patients	57	67.6 (24.4)
Louis et al. (49)	US	ET patients	121	53.6 (25.9)
Louis et al. (59)	US	*ET patients – 0–9 years with tremor*	*96*	*45.6 (31.7)*
	US	*ET patients – 10–19 years with tremor*	*69*	*49.6 (29.8)*
	US	*ET patients – 20–29 years with tremor*	*62*	*58.2 (25.7)*
	US	*ET patients – 30–39 years with tremor*	*34*	*57.8 (27.5)*
	US	*ET patients – ≥ 40 years with tremor*	*74*	*60.6 (27.5)*
Monin et al.^*^^†^ (30)	US	ET patients	50	14.2 (2.8)
Morgan et al.^*^^†^ (31)	US	ET patients	55	14.2 (4.9)

*CUDQET, Columbia University Disability Questionnaire for Essential Tremor; ET, Essential tremor; SD, Standard deviation; US, United States.*

* *Indicates a variation of the CUDQET scored out of 20 was used.*

† *Studies recruited patients from the same ongoing longitudinal COGNET study.*

*Population in italics refers to subgroups of ET patients reported in the study*.

In studies using QUEST, patients with ET consistently experienced high levels of impairment (i.e., score of 4 on each item or indicated they “always” have interference in activities because of ET) on the physical and work/finance subscales including writing (30.1–34.8%), drinking (18.4–40.0%), fixing small things around the house (13.6–39.6%), and performing their job (8.5–46.0%) (51, 56). Patients also reported moderate levels of impairment (i.e., a score of 2 or 3 on each item indicating they had some or frequent impairment because of ET) on the subscales for writing (50.5%), drinking (50.5%), eating (46.6%), and fixing small things (41.8%) (51). The QUEST physical sub-score was significantly correlated with greater tremor severity and longer tremor duration, which may result in increased inability to do tasks (51).

Additionally, one study assessed disability using the SIS and reported that patients with ET felt significant levels of incompetence as demonstrated by low mean SIS scores (26). Functional activities impacted by ET assessed using the FAQ showed that patients with ET had statistically significantly more difficulty compared to controls in performing a variety of cognitive functions, including paying attention to, understanding, or discussing a television show, book, or magazine; remembering appointments, family occasions, holidays, and to take medications; and greeting people appropriately (40).

#### Impact on HRQOL

Direct measures of HRQOL were reported in 10 publications (23, 34, 45, 47–49, 51, 52, 55, 56) conducted in the US (34, 49, 51), Europe (23, 48, 52), Asia (47, 55, 56), and Africa (45). The mean age across studies reporting HRQOL varied from 25 to 75 years (23, 34, 45, 47–49, 51, 52, 55, 56). Impact on HRQOL was assessed using the QUEST summary index (QSI), comprised of the mean of the five QUEST subscales assessing the impact of ET and tremors on physical, communication, work/finance, psychosocial, and hobbies/leisure domains in six publications (34, 47, 49, 51, 55, 56). Impact on HRQOL was also assessed using physical and mental component scores where higher scores indicate better HRQOL in the 36-item Short Form Survey (SF-36) in three publications (23, 45, 48), and the SF-12 in one publication (52).

Patients with ET demonstrated lower HRQOL based on scores of disease agnostic and ET-specific instruments. Mean QSI values were reported for all patients with ET in four publications and ranged from 17.1 to 24.2 (scored between 0 and 100 with higher scores indicating lower HRQOL; Table 4) (47, 51, 55, 56). According to work by Kovács et al. (61), mean QSI scores greater than 11.25 indicate clinically meaningful disability and greater than 20.35 indicate severe disability. Based on these thresholds, many patients with ET from these studies experience moderate to severe disability (47, 51, 55, 56). Additionally, QUEST total scores were significantly correlated with total tremor score (34, 56). In the three publications reporting data using the SF-36, two studies reported patients with ET had statistically significantly lower total mental component scores compared to controls (Table 5) (23, 45, 48). Statistically significantly lower physical component scores were reported in two of three publications (45, 48). Mental health domain sub-scores were significantly lower for older patients with ET compared to younger patients (45). HRQOL was also demonstrated to be significantly reduced in patients who had voice tremor or lower limb tremor (34, 56).

**Table 4:** Mean Quality of Life in Essential Tremor Questionnaire scores.

**Publication**	**Country**	**Population**	**Sample size**	**QUEST score/subscore (if** **available)**	**Mean (SD)**
Chandran and Pal^‡^^†^ (56)	India	ET patients	50	Total score	24.2 (19.2)
				Communication subscore	23.9 (36.9)
				Hobbies/leisure subscore	6.8 (17.3)
				Physical subscore	29.3 (26.7)
				Psychosocial subscore	36.4 (28.7)
				Work/finance subscore	23.5 (29.9)
Chandran et al.^‡^^†^ (55)	India	ET patients	50	Total score	24.2 (19.2)
Louis et al.^δ^ (34)	US	*ET patients – minimal depressive symptoms*	*41*	*Total score*	*22.1 (16.5)*
		*ET patients – moderate depressive symptoms*	*24*	*Total score*	*37.1 (17.3)*
		*ET patients – severe depressive symptoms*	*5*	*Total score*	*48.4 (24.2)*
Louis and Machado^α^ (51)	US	ET patients	103	Total score	19.0 (16.2)
				Communication subscore	10.2 (16.9)
				Hobbies/leisure subscore	18.4 (33.0)
				Physical subscore	39.0 (25.1)
				Psychosocial subscore	22.0 (19.8)
				Work/finance subscore	7.9 (15.1)
Peng et al. (47)	China	ET patients	199	Total score	17.1 (15.5)

*Population in italics refers to subgroups of ET patients reported in the study.*

*ET, Essential tremor; QUEST, Quality of Life in Essential Tremor Questionnaire; SD, Standard deviation; US, United States.*

† *Studies reported mean total scores as a quest summary index (QSI), which is computed by calculating the mean of each of the five QUEST subscales. A higher score indicates greater disability.*

δ *QUEST total score is calculated based on 26 of the 30 items contained in the QUEST, given four items from the work/finance domain were not applicable to the vast majority of the study population who were elderly and past retirement.*

α *26-item QUEST scores also available in publication.*

‡ *Studies recruited patients from the same site in India*.

**Table 5:** Mean scores for the 36-item short form survey.

**Publication**	**Country**	**Population**	**Sample size**	**PCS mean (SD)**	**MCS mean (SD)**
Lorenz et al.^α^ (52)	Germany	OPC ET patients	107	43.7 (9.6)^*^	49.6 (11.5)^*^
		CBC ET patients	90	48.1 (9.8)^*^	51.8 (8.4)^*^
Musacchio et al.^δ^ (48)	Germany	ET patients	110	46.2 (10.3)	45.9 (11.1)
		General German population	N/A	50.2 (10.2)	51.5 (8.1)
Sengul et al. (23)	Turkey	Young ET patients	45	48.7 (8.9)	38.7 (8.9)
		Young controls	35	52.2 (6.7)	44.1 (10.5)
Shalash et al.^**^^†^^δ^ (45)	Egypt	ET patients	30	NR	NR
		Controls	30	NR	NR

*CBC, Community-based cohort; ET, Essential tremor; MCS, Mental Component Score; OPC, Outpatient Cohort; PCS, Physical Component Score; SD, Standard deviation; SF-12, 12-item Short Form Survey; SF-36, 36-item Short Form Survey.*

* *Mean scores re.ported for the 12-item Short Form Survey (SF-12), not the 36-item Short Form Survey (SF-36).*

** *SF-36 mental and physical component scores were not available; only the scores for domains were reported.*

† *Scores for each domain were also reported for young and old ET patients and controls.*

δ *Scores for each domain that encompass the mental and physical component scores also available.*

α *Matched ET patients and controls (38 in each cohort) were included for the SF-36 analysis; N = 107 OPC ET patients and N = 90 CBC ET patients were included in the study*.

## Discussion

This review is, to the best of our knowledge, the first comprehensive and systematic synthesis of literature covering the clinical and humanistic burden of ET, beyond the tremor itself. The findings demonstrate that patients experience myriad manifestations beyond tremor, including physical issues, such as difficulty walking, balance issues, and propensity to fall, and non-physical issues, such as mood disorders, fatigue, sleep disturbances, and cognitive impairment. Taken together, these manifestations can have significant deleterious effects on patients' independence, psychosocial experiences, and overall HRQOL. Although the presentation of non-tremor manifestations and/or consequences of ET is heterogeneous in nature, patients typically experience more than one manifestation, contributing to higher morbidity and also substantive costs related to disease management (62).

Prior examinations of ET have not provided comprehensive assessments of the burdens experienced by patients with ET and consequently may underestimate the burden of ET. Existing literature often focuses on a single dimension of disease burden, possibly to ensure clarity in research design and endpoints measured, which siloes research. On rare occasions when studies measure multiple manifestations and/or consequences of the disease, the studies often include those that are highly correlated with each other (e.g., activities of daily living and depression, or depression and enfeeblement) (11, 25, 26). Such unidimensional assessments may not provide the complete picture of the true, multi-dimensional burden of ET. A prior review article succinctly summarized the state of the field in ET in terms of identification of non-motor symptoms. The understanding of the variable nature of ET manifestations in the previous review is well-aligned with this current work (63).

Although ET is often considered a disease of older patients, in reality, it has a bimodal onset, with some patients developing ET in early adulthood (7). Onset in early adulthood has the potential to impact work productivity. Notably, with early adulthood onset ET, the severity of tremor and disability slowly increases over time and approximately a quarter of patients required occupation changes or retirement (10, 64). This review found limited recent evidence for the impact of ET on work productivity. The work/finances sub-score of QUEST was evaluated in some studies, but no other data using validated work productivity instruments were identified in the current search period (34, 51, 56). The paucity of available data makes it difficult to fully assess the impact of ET on work productivity, highlighting a potential area for future study.

In addition to work productivity, decreased capacity to perform ADL due to the manifestations of ET may be measured using either ET-specific instruments, such as the QUEST, or generic, disease-agnostic instruments often used in older adults like the Lawton IADL (65, 66). ET-specific instruments are more likely to detect and adequately assess the inability to perform tasks affected by mild to severe tremors. As a result, these instruments may be more sensitive to the true impact on ADL for patients with ET than the more commonly used, generic scales. For example, Lawton IADL scores reported in the included studies showed patients were relatively independent in functional tasks such as cooking, cleaning, etc.; however, these data could be misleading (25–27). Instrumental ADL involve tasks that help an individual live in a community, but ET patients are more hindered in performing basic ADL involving self-care, and activities that require fine motor skills, so a broad IADL scale may not be adequately focused on the tasks patients feel less comfortable/capable of doing independently (67). In contrast, publications reporting disability due to tremor using CUDQET, and impact on ADL using QUEST, captured the impact of ET on more nuanced daily activities more susceptible to tremor including brushing, flossing, holding items, dressing oneself, drinking, and other necessary personal chores crucial for independent functioning. The questions in ET-specific scales directly ask patients what level of inability tremors have caused in the performance of such ADL (34, 47, 49, 51, 55, 56). Like these scales, other ET-specific scales should capture how other manifestations in ET (e.g., imbalance, embarrassment) may also cause an inability to perform ADL.

Finally, in addition to demonstrating the multi-dimensional burden of ET, this review identified several avenues of future research and evidence gaps to be addressed. First, it is worth noting that a diagnostic classification of “ET plus” has been proposed for cases in which the clinical manifestation of ET includes other mild neurologic signs of unknown clinical significance and some of the features reviewed herein (i.e., cognitive decline and motor symptoms, such as impaired gait and questionable dystonia) (5). However, there is controversy surrounding the use of ET plus as a diagnostic label (68–70). Much work remains to be done in terms of defining how various neurologic features arise at different points throughout the ET disease course and whether the emergence of such symptoms, in fact, represents an entity that is distinguishable from ET. Whether ET plus is a disease subtype per se, or instead a more advanced disease stage of ET is unclear; recent work suggests that features of ET plus are both age- and stage-dependent (68, 70). Whether ET plus is a stage or a subtype, it is clear that the presence of additional clinical features has important implications for patients in everyday life.

Second, a report in 2015 described a lack of overall disease awareness and tools that prevents physicians and patients from effectively communicating the burden associated with ET to each other, which may inhibit the delivery of optimal care (71). In line with this idea, the current review highlighted gaps in the evidence including relatively few studies that have characterized how different manifestations of ET evolve over time. While current research has used a multitude of available instruments and diagnostic techniques to continue to evaluate patients with ET and their clinical profile, there are certainly limitations to how accurately these assessments represent the patients. This gap may reflect a lack of standardized approaches to evaluate ET-specific symptoms and the fact that researchers often rely on generic scales, likely because these measures are established and/or validated. However, they may not be specific enough to adequately capture the multi-dimensional burden of ET. For many symptoms of ET, like depression, there exists a wide variety of instruments, but there is a lack of consensus or validation within ET cohorts on which of these instruments best capture the patient's lived experience with ET. Alternately, for research using newer, ET-specific scales, there is little guidance on which measurements are best suited to capture ET's true burden. In addition to addressing these areas, more research is needed to specify aspects of functional impairment including how ADL may differ in relation to clinical heterogeneity in ET, and how productivity is impacted among individuals in the workforce. For example, understanding whether ET impacts only specific tasks at work or whether it causes patients to seek early retirement, shift to part-time work, or stay employed with lower work-productivity are important factors to examine.

Third, work and daily activities can also be seriously impacted by cognitive impairment. More research is needed to understand the domains of cognitive impairment that are most impacted due to ET. The specific cognitive domains affected by ET, and the mechanisms by which cognition becomes impaired, are likely to be heterogenous (12, 72). Most conceptualizations of cognitive impairment in ET have focused on a dysexecutive syndrome believed to reflect compromised fronto-cerebellar networks; however, there is increasing recognition of memory deficits in ET which may implicate hippocampal involvement. While a detailed discussion of the evidence for heterogeneous contributors to cognitive impairment is beyond the scope of this review; a recent review addresses this topic in detail (72).

Lastly, there was an overall lack of adequate research designed to focus primarily on the humanistic burden of ET. Issues relating to emotions and feelings, such as embarrassment and alexithymia, can make social interactions very challenging for patients, potentially leading to isolation, depression, etc., which in turn create additional burdens. More robust assessments of such burden can help patients receive appropriate specialist care, additional pharmacological therapy, counseling, and behavioral therapy to combat these challenges.

### Limitations

This review has certain methodological limitations that should be considered when contextualizing this summary of evidence. This review focused on the last 10 years of relevant published literature on ET to ensure that the studies captured reflect the latest advances in the diagnosis, assessment, classification, and treatment of patients. This approach excludes any studies published further in the past, but the findings from this review are consistent with those of a previously published literature review (63).

Multiple publications included participants drawn from the same large prospective study (i.e., the COGNET study) (24–32). Although these publications cover different enrollment periods or apply different eligibility criteria, it is likely that some patients are double counted across these related publications. However, as there were no quantitative analyses performed, the influence on the findings in this review should be limited.

The studies included in this review focused on the use of specific structured questionnaires for the assessment of severity of manifestations beyond tremor. However, there are other methods of evaluating manifestations of ET, such as use of kinematic analysis for movement-related manifestations and diagnostic tools like the Structured Clinical Interview for DSM-5 for identification of mental health conditions including depression/anxiety in patients (73, 74). This review is not an exhaustive evaluation of non-motor manifestations that may significantly contribute to the burden of illness in ET; conditions such as autonomic dysfunction, hearing loss, and olfactory dysfunction should be considered in the assessment of these patients and studied in greater detail (63, 75, 76).

This review does not focus or synthesize evidence on the pathophysiology or mechanisms of the dysfunctions seen in patients with ET. For example, potential cerebellar involvement could partially explain non-motor manifestations such as cognitive, autonomic, and sensory deficits (63). Having knowledge of these mechanisms could help predict or understand the non-motor manifestations of ET. This review provides a brief look at some differences in manifestations between certain subgroups of patients, such as old vs. young patients or patients with midline vs. head tremor. Patients with midline tremor were found to be more likely to have severe cognition, sleep, and fatigue-related outcomes and should be evaluated more closely in future research (46, 47).

Additionally, publications addressing the manifestations of interest were excluded if they did not report quantitative data from individual studies. This approach was necessary to facilitate qualitative comparisons between studies. Most included studies were also retrospective or cross-sectional in nature and could not establish causal relationships between clinical variables. This review was also not designed to evaluate caregiver burden, but studies have reported on this important issue that adds to the overall burden of ET (25, 28, 31). One study reported that caregivers experienced higher burden with assistance in performing tasks, high caregiving hours per week, and long duration of continued caregiving, but these measures were not associated with the patient's tremor severity or disability score (31). Caregivers most often provided support with writing tasks for patients and around 11% of caregivers provided 25–40 h per weeks of support (31). These prior studies highlight an important gap that should be addressed by future research. Finally, only a limited number of studies explored correlations between different manifestations and/or consequences of ET with specific features of ET (e.g., tremor severity, age of onset), and the findings generally lacked independent verification, further highlighting the lack of cohesive studies conducted in this disease area.

## Conclusions

Essential tremor is a debilitating, chronic condition with physical and mental manifestations that extend well beyond motor function, with grave impact for patients' ADL and HRQOL. Although there is a significant body of published evidence on the outcomes of ET, current research tends to be siloed, focusing on specific, narrow outcome measures. There is a lack of literature on the multifaceted nature of the disease, and as a result, the comprehensive burden of disease experienced by patients is likely underestimated. This review provides a first synthesis of existing literature on non-tremor manifestations and/or consequences of ET to better demonstrate the full burden of disease from the clinical and humanistic perspectives and highlight gaps in our understanding. Future research is required to further define the multi-dimensional aspects of ET, its impact on patients, and how the appropriate treatment and management of ET can improve patients' lives.

## Data availability statement

The original contributions presented in the study are included in the article/supplementary material, further inquiries can be directed to the corresponding author/s.

## Author contributions

MG and AE-L were the principal study investigators. MG, AE-L, SN, KH, NH, and SC designed the study. SN, SR, KH, and NH were responsible for execution of the study. All authors were involved in the interpretation of the data as well as writing the manuscript. All authors contributed to the article and approved the submitted version.

## Funding

This study received funding from Sage Therapeutics, Inc. and Biogen. The funders were not involved in the study design, collection, analysis, interpretation of data, the writing of this article or the decision to submit it for publication.

## Conflict of interest

MG is an employee of Sage Therapeutics, Inc. and AE-L was an employee of Sage Therapeutics, Inc. at the time of conducting this research. Both own stock/stock options. SN, SR, KH, and NH were contracted to conduct this research on behalf of Sage Therapeutics. SN and SR were employees of Trinity Life Sciences at the time of conducting this research. KH and NH are current employees of Trinity Life Sciences. SC is a paid consultant for Sage Therapeutics, Inc. and the Association for Frontotemporal Dementia.

## Publisher's note

All claims expressed in this article are solely those of the authors and do not necessarily represent those of their affiliated organizations, or those of the publisher, the editors and the reviewers. Any product that may be evaluated in this article, or claim that may be made by its manufacturer, is not guaranteed or endorsed by the publisher.
