# Supplementary material for: Essential tremor patients experience significant burden beyond tremor: A systematic literature review
Source: Front Neurol. 2022 Jul 22;13:891446. doi: 10.3389/fneur.2022.891446 (PMC9354397; doi:10.3389/fneur.2022.891446)
Supplement: Supplementary file 1 [file Table_1.DOCX]

Supplementary Material

# Supplementary Data

Supplementary Table 1. Electronic Database Search Terms

| PubMed (via pubmed.gov) | | |
| --- | --- | --- |
| **Search No.** | **Type of Term** | **Search String** |
| 1 | Disease terms | ("essential tremor"[Mesh] OR ("essential tremor"[Title/Abstract] OR "kinetic tremor"[Title/Abstract] OR "benign essential tremor"[Title/Abstract] OR "familial tremor"[Title/Abstract] OR "hereditary essential tremor"[Title/Abstract])) |
| 2 | Publication type limits | "Comment"[pt] OR "Editorial"[pt] OR "Letter"[pt] OR "News"[pt] OR "Published Erratum"[pt] OR "Retraction of Publication"[pt] OR "Case Reports"[pt] OR "Biography"[pt] OR "Expression of Concern"[pt] OR "Patient Education Handout"[pt] OR "Directory"[pt] OR "Newspaper Article"[pt] OR "Historical Article"[pt] OR "Retracted Publication"[pt] OR commentary[ti] OR editorial[ti] OR "letter to editor"[ti] OR erratum[ti] OR correction[ti] OR congresses as topic [mesh] OR (animals[mh] not (animals[mh] and humans[mh])) |
| **Embase (via embase.com)** | | |
| **Search No.** | **Type of Term** | **Search String** |
| 1 | Disease terms | 'essential tremor'/exp OR 'essential tremor':ti,ab OR 'kinetic tremor':ti,ab OR 'benign essential tremor':ti,ab OR 'familial tremor':ti,ab OR 'hereditary essential tremor':ti,ab |
| 2 | Publication type limits | 'editorial'/exp OR 'book'/exp OR 'erratum'/exp OR 'letter'/exp OR 'note'/exp OR 'conference paper'/exp OR 'in vitro study'/exp OR 'in vivo study'/exp OR 'cell culture'/exp OR 'cell line'/exp OR 'mathematical model'/exp OR 'theoretical model'/exp OR 'feasibility study'/exp OR 'pilot study'/exp OR [editorial]/lim OR [erratum]/lim OR [letter]/lim OR [note]/lim OR ('animal'/exp NOT ('animal'/exp AND 'human'/exp)) |
| **CENTRAL and CDSR (via Cochrane Library)** | | |
| **Search No.** | **Type of Term** | **Search String** |
| 1 | Disease terms | MeSH descriptor: [Essential Tremor] explode all trees OR ("essential tremor" OR "kinetic tremor" OR "benign essential tremor" OR "familial tremor" OR "hereditary essential tremor"):ti,ab,kw |

Supplementary Table 2. Study Eligibility Criteria

| Parameter | Inclusion Criteria | Exclusion Criteria |
| --- | --- | --- |
| Population | Adult patients diagnosed with ET, not restricted by age of onset so including familial ET | Other movement disorders or neurodegenerative diseases  Studies with mix of movement disorder patients, but not reporting data for ET patients separately |
| Intervention/Comparator | None | None |
| Outcomes | Outcomes measured using structured instruments/questionnaires for the assessment of outcome categories: gait, balance, and falls, cognitive impairment, depression and anxiety, sleep disturbances and fatigue, impact on activities of daily living, impact on health-related quality of life, and psychosocial impact | Epidemiology, efficacy and safety of treatments, healthcare resource use and outcomes, treatment patterns |
| Study Design | Observational, non-randomized studies such as claims analysis, registry studies, electronic medical chart review | Preclinical studies, genetic models, animal studies, case reports, case studies, randomized controlled trials, economic evaluations |

Supplementary Table 3. Summary of Study and Patient Characteristics of Included Publications

| First Author, Year | Study Country | Data Source | Study Population | Sample Size | Age - Mean (SD) *(in years)* | Age of Onset - Mean (SD) *(in years)* | Disease Duration - Mean (SD) *(in years)* | Disease Severity - Mean (SD) | Disease Severity Criteria | Family History of ET |
| --- | --- | --- | --- | --- | --- | --- | --- | --- | --- | --- |
| Acar, 2019 (1) | Turkey | Hospital Cohort | Controls | 38 | 33.2 (10.1) | N/A | N/A | N/A | N/A | NR |
|  |  |  | ET patients | 40 | 33.6 (12.2) | NR | NR | NR | NR | NR |
| Cersonsky, 2019 (a) (2) | US | COGNET | ET patients | 98 | 80.5 (9.0) | N/A | 41.6 (22.8) | 20.7 (5.6) | WHIGETTRS | NR |
|  | US | COGNET | ET caregivers | 98 | 71.0 (11.8) | N/A | N/A | N/A | N/A | NR |
| Cersonsky, 2019 (b) (3) | US | COGNET | ET patients | 60 | 70.2 (6.8) | NR | 37.2 (18.9) | 19.4 (4.3) | WHIGETTRS | NR |
| Chandran, 2013 (4) | India | Hospital Cohort | ET patients | 50 | 40.7 (16.2) | 32.2 (18.9) | 8.4 (10.0) | 16.8 (8.7) | Modified FTMTRS | 58.0% |
| Chandran, 2012 (5) | India | Hospital Cohort | ET patients | 50 | 40.7 (16.2) | 32.2 (18.9) | 8.4 (10.0) | 26.6 (16.2) | FTMTRS | 58.0% |
|  | India | Hospital Cohort | Controls | 50 | 42.3 (15.3) | N/A | N/A | N/A | N/A | 0.0% |
| Cinar, 2013 (6) | Turkey | Hospital Cohort | ET patients | 90 | 61.4 (17) | NR | 6.7 (4.5) | 22.3 (14) | FTMTRS | NR |
|  |  |  | Controls | 50 | 60.9 (15) | N/A | N/A | N/A | N/A | NR |
| Guiterrez, 2016 (7) | US | ETCBR | ET patients | 164 | 83.3 (5.6) | 42.6 (23.0) | 40.7 (22.5) | NR | NR | 25.6% |
| Huang, 2019 (8) | China | Hospital Cohort | ET patients | 245 | 53.3 (16.4) | 43.4 (17.8) | 9.87 (9.74) | NR | NR | 42.0% |
| Huang, 2020 (9) | China | Hospital Cohort | ET patients | 290 | 53.74 (15.78) | 43.93 (17.15) | 9.77 (9.53) | NR | NR | 58.30% |
| Huey, 2018 (10) | US | COGNET | ET patients | 233 | 79.0 (9.5) | NR | NR | 23.9 (5.6) | WHIGETTRS | NR |
| Kellner, 2017 (11) | US | COGNET | ET patients | 57 | 77.2 (10.2) | 40.9 (22.0) | 36.2 (21.6) | 23.1 (5.6) | WHIGETTRS | NR |
|  | US | COGNET | ET caregivers | 57 | 66.6 (12.7) | N/A | N/A | N/A | N/A | 31.4% |
| Kudrevatykh, 2020 (12) | Russia | Hospital Cohort | ET patients | 55 | 71 (45-90)* | NR | 7 (1-50)* | 8.0 (2.0-23.0)* | TETRAS | NR |
| Lacerte, 2014 (13) | Canada | Survey | ET patients | 46 | 57.2 (19.5) | 31.5 (22.6) | 24.9 (19.1) | NR | NR | NR |
| Lee, 2015 (14) | South Korea | Hospital Cohort | ET patients | 60 | 52.5 (16.9) | NR | 10.1 (8.2) | NR | NR | NR |
|  | Korea | Hospital Cohort | Controls | 22 | 65.9 (17.5) | N/A | N/A | N/A | N/A | NR |
| Lorenz, 2011 (15) | Germany | Hospital Cohort | ET patients -- Outpatient Cohort | 107 | 63.1 (15.8) | 32.7 (20.7) | 27.6 (18.7) | 24.7 (13.4) | FTMTRS | 61.7% |
|  | Germany | Hospital Cohort | ET patients -- Community-based cohort | 90 | 72.1 (6.4) | 56.3 (16.1) | 16.3 (13.8) | 18.5 (9.3) | FTMTRS | 46.7% |
| Louis, 2010 (a) (16) | Spain | NEDICES | ET patients | 135 | 73.6 (6.3) | 63.5 (13.9) | NR | NR | NR | 40.0% |
|  | Spain | NEDICES | Controls | 2184 | 72.4 (5.8) | N/A | N/A | N/A | N/A | NR |
| Louis, 2010 (b) (17) | Spain | NEDICES | ET patients | 208 | 75.1 (6.9) | NR | 9.6 (11.4) | NR | NR | NR |
|  | Spain | NEDICES | Controls | 3616 | 73.5 (6.4) | N/A | N/A | N/A | N/A | NR |
| Louis, 2012 (a) (18) | US | CMUC Cohort | ET patients – Minimal depressive symptoms | 41 | 73.1 (11.1) | NR | 30.6 (17.6) | 21.9 (6.0) | WHIGETTRS | NR |
|  |  |  | ET patients – Moderate depressive symptoms | 24 | 66.8 (16.1) | NR | 36.0 (18.2) | 20.7 (5.3) | WHIGETTRS | NR |
|  |  |  | ET patients – Severe depressive symptoms | 5 | 75.0 (10.1) | NR | 25.2 (11.1) | 18.8 (3.8) | WHIGETTRS | NR |
| Louis, 2012 (b) (19) | US | CMUC Cohort | ET patients | 79 | 70.8 (13.4) | NR | NR | NR | NR | NR |
|  |  |  | Controls | 80 | 70.8 (9.6) | N/A | N/A | N/A | N/A | NR |
| Louis, 2012 (c) (20) | US | CMUC Cohort | ET patients | 59 | 71.2 (14.6) | 38.1 (20.6) | 33.2 (18.0) | 20.7 (5.0) | WHIGETTRS | 62.7% |
|  |  |  | Controls | 82 | 71.6 (10.8) | N/A | N/A | N/A | N/A | NR |
| Louis, 2012 (d) (21) | US | CMUC Cohort | ET patients | 161 | 83.9 (5.7) | 42.1 (22.7) | 41.6 (21.9) | 24.7 (7.5) | CIRS | NR |
| Louis, 2013 (22) | US | CMUC Cohort | ET patients | 335 | 68.4 (14.5) | 44.5 (22.7) | 23.9 (18.8) | 19.6 (6.9) | WHIGETTRS | NR |
| Louis, 2015 (23) | US | CMUC Cohort | ET patients | 103 | 69.7 (12.3) | 39.1 (19.3) | 30.9 (17.3) | 20.5 (5.6) | WHIGETTRS | 28.2% |
| Louis, 2016 (a) (24) | US | CMUC Cohort | ET patients | 121 | 69.9 (12.6) | 38.2 (19.5) | 31.7 (18.3) | 20.8 (59.6) | WHIGETTRS | 28.1% |
| Louis, 2016 (b) (25) | US | COGNET | ET patients | 100 | 80.5 (8.1) | 39.1 (21.2) | NR | 21.5 (5.8) | WHIGETTRS | 50.0% |
| Louis, 2016 (c) (26) | US | CMUC Cohort | ET patients | 91 | 70.4 (12.8) | 37.7 (18.1) | 36.9 (18.7) | 20.6 (5.9) | WHIGETTRS | 30.8% |
| Manorenj, 2019 (27) | India | Hospital Cohort | ET patients | 45 | 44.0 (15.0) | 40.6 (15.4) | 6 (2.81) | 18.66 (9.67) | TETRAS | 35.0% |
| Monin, 2017 (28) | US | COGNET | ET patients | 50 | 76.8 (10.6) | 42.1 (22.1) | 34.7 (21.0) | 20.5 (5.6) | WHIGETTRS | 46.0% |
|  |  |  | Close others | 50 | 65.4 (12.5) | N/A | N/A | N/A | N/A | NR |
| Morgan, 2017 (29) | US | COGNET | ET patients | 55 | 76.9 (10.1) | 41.4 (22.3) | 35.5 (21.5) | 23.1 (5.5) | WHIGETTRS | NR |
|  |  |  | ET caregivers | 55 | 66.6 (12.7) | N/A | N/A | N/A | N/A | 28.2% |
| Musacchio, 2016 (30) | Germany | Hospital Cohort | ET patients | 110 | 65.2 (14.1) | 41.4 (21.0) | 23.8 (16.4) | 36.8 (14.6) | FTMTRS | 74.5% |
| Peng, 2020 (31) | China | Hospital Cohort | ET patients | 199 | 56.17 (14.464) | 43.24 (15.461) | 12.93 (7.680) | 26.79 (16.075) | FTMTRS | 56.28% |
| Rao, 2011 (32) | US | CMUC Cohort | ET patients | 104 | 86.0 (4.6) | 43.3 (23.6) | NR | NR | NR | NR |
|  |  |  | Controls | 40 | 84.1 (4.2) | N/A | N/A | N/A | N/A | NR |
| Rao, 2014 (33) | US | CMUC Cohort | ET patients -- Low Cognition | 63 | 84.9 (5.7) | 43.3 (23.4) | NR | NR | NR | NR |
|  |  |  | ET patients-- High Cognition | 69 | 82.5 (5.6) | 40.1 (21.8) | NR | NR | NR | NR |
|  |  |  | Controls | 48 | 79.5 (6.7) | N/A | N/A | N/A | N/A | NR |
| Rohl, 2016 (34) | US | COGNET | ET patients -- Normal Cognition | 67 | 79.9 (9.1) | NR | 35.6 (20.4) | 21.2 (5.9) | WHIGETTRS | NR |
|  |  |  | ET patients -- Mild Cognitive Impairment | 16 | 84.5 (7.0) | NR | 38.8 (22.4) | 21.3 (6.4) | WHIGETTRS | NR |
|  |  |  | ET patients -- Dementia | 13 | 87.2 (6.4) | NR | 48.8 (25.3) | 25.2 (8.1) | WHIGETTRS | NR |
| Sengul, 2015 (35) | Turkey | Hospital Cohort | ET patients | 45 | 24.6 (7.2) | NR | 4.36 (3.94) | 10.2 (4.2) | FTMTRS | 22.2% |
|  | Turkey |  | Controls | 35 | 24.8 (5.4) | N/A | N/A | N/A | N/A | NR |
| Sengul, 2020 (36) | Turkey | Hospital Cohort | ET patients | 100 | 43.5 (20.4) | NR | 9.6 (11.0) | 23.3 (12.5) | FTMTRS | 68.0% |
|  |  |  | Controls | 100 | 40.8 (11.3) | N/A | N/A | N/A | N/A | NR |
| Shalash, 2019 (37) | Egypt | Hospital Cohort | ET patients | 30 | 45.20 (18.10) | 34.73 (16.56) | 10.40 (7.86) | 42.10 (13.80) | FTMTRS | 42.50% |
|  |  |  | Controls | 30 | 43.43 (17.27) | N/A | N/A | N/A | N/A | NR |
| Smeltere, 2017 (38) | Latvia | Hospital Cohort | ET patients | 40 | 52.1 (20.2) | NR | NR | 29.0 (20.1) | FTMTRS | NR |
|  |  |  | Controls | 39 | 55.9 (16.4) | N/A | N/A | N/A | N/A | NR |
| Zubair, 2018 (39) | US | COGNET | ET patients | 141 | 81.1 (8.8) | 40.5 (23.0) | NR | 21.1 (6.0) | WHIGETTRS | NR |

*Data reported as median (range)

**Abbreviations:** CIRS = Cumulative Illness Rating Scale; COGNET = Clinical Pathological Study of Cognitive Impairment in Essential Tremor; CMUC Cohort = Clinical Epidemiological Research Study at Columbia University Medical Center; ET = Essential tremor; ETCBR = Essential Tremor Centralized Brain Repository Future Donors; FTMTRS = Fahn-Tolosa-Marin Tremor Rating Scale; N/A = Not applicable; NEDICES = Neurological Disorder of Central Spain Epidemiological Study; NR = Not reported; SD = Standard deviation; TETRAS = The Essential Tremor Rating Assessment Scale; US = United States; WHIGETTRS = Washington Heights-Inwood Genetic Study of ET Tremor Rating Scale

# References for Supplementary Data

1. Acar BA, Acar T. Essential Tremor is not Only a Movement Disorder; Its Relationship with Sleep and Anxiety. *Noro Psikiyatr Ars* (2019) 56(1):18-22. Epub 2019/03/27. doi: 10.5152/npa.2017.22858. PubMed PMID: 30911232; PubMed Central PMCID: PMCPMC6427083.

2. Cersonsky TEK, Diaz DT, Kellner S, Hickman R, Zdrodowska MA, Monin JK, et al. Enfeeblement in Elders with Essential Tremor: Characterizing the Phenomenon and Its Role in Caregiver Burden. *Tremor Other Hyperkinet Mov (N Y)* (2019) 9:10.7916/tohm.v0.687. doi: 10.7916/tohm.v0.687. PubMed PMID: 31709127.

3. Cersonsky TEK, Kellner S, Morgan S, Cosentino S, Koo BB, de Figueiredo JM, et al. Demoralization in essential tremor: prevalence, clinical correlates, and dissociation from tremor severity. *CNS Spectr* (2020) 25(1):16-23. Epub 2019/04/04. doi: 10.1017/s1092852918001633. PubMed PMID: 30940264.

4. Chandran V, Pal PK. Quality of life and its determinants in essential tremor. *Parkinsonism Relat Disord* (2013) 19(1):62-5. Epub 2012/07/10. doi: 10.1016/j.parkreldis.2012.06.011. PubMed PMID: 22771281.

5. Chandran V, Pal PK, Reddy JY, Thennarasu K, Yadav R, Shivashankar N. Non-motor features in essential tremor. *Acta Neurol Scand* (2012) 125(5):332-7. Epub 2011/07/23. doi: 10.1111/j.1600-0404.2011.01573.x. PubMed PMID: 21777207.

6. Cinar N, Sahin S, Okluoglu Onay T, Karsidag S. Balance in essential tremor during tandem gait: is the first mis-step an important finding? *J Clin Neurosci* (2013) 20(10):1433-7. Epub 2013/08/21. doi: 10.1016/j.jocn.2013.01.013. PubMed PMID: 23953429.

7. Gutierrez J, Park J, Badejo O, Louis ED. Worse and Worse and Worse: Essential Tremor Patients' Longitudinal Perspectives on Their Condition. *Front Neurol* (2016) 7:175-. doi: 10.3389/fneur.2016.00175. PubMed PMID: 27790185.

8. Huang H, Yang X, Zhao Q, Chen Y, Ning P, Shen Q, et al. Prevalence and Risk Factors of Depression and Anxiety in Essential Tremor Patients: A Cross-Sectional Study in Southwest China. *Front Neurol* (2019) 10:1194. Epub 2019/12/06. doi: 10.3389/fneur.2019.01194. PubMed PMID: 31803131; PubMed Central PMCID: PMCPMC6873801.

9. Huang HY, Zhao QZ, Ning PP, Shen QY, Wang H, Xie D, et al. Non-motor symptoms are associated with midline tremor in essential tremor. *Acta Neurol Scand* (2020) 142(5):501-10. Epub 2020/06/02. doi: 10.1111/ane.13290. PubMed PMID: 32476139.

10. Huey ED, Cosentino S, Chapman S, Azar M, Rohl B, Collins K, et al. Self-report depressive symptoms are dissociated from tremor severity in essential tremor. *Parkinsonism & related disorders* (2018) 50:87-93. Epub 2018/02/20. doi: 10.1016/j.parkreldis.2018.02.031. PubMed PMID: 29499915.

11. Kellner S, Morgan S, Gutierrez J, Collins K, Rohl B, Migliore F, et al. Perceived embarrassment and caregiver burden in essential tremor caregivers. *J Neurol Sci* (2017) 383:205-10. Epub 2017/11/20. doi: 10.1016/j.jns.2017.11.020. PubMed PMID: 29246614.

12. Kudrevatykh A, Senkevich K, Miliukhina I. Postural instability and neuropsychiatric disturbance in the overlapping phenotype of essential tremor and Parkinson's Disease. *Neurophysiol Clin* (2020) 50(6):489-94. Epub 2020/09/03. doi: 10.1016/j.neucli.2020.07.001. PubMed PMID: 32873435.

13. Lacerte A, Chouinard S, Jodoin N, Bernard G, Rouleau GA, Panisset M. Increased Prevalence of Non-motor Symptoms in Essential Tremor. *Tremor Other Hyperkinet Mov (N Y)* (2014) 4:162. Epub 2014/09/24. doi: 10.7916/d82v2d91. PubMed PMID: 25247108; PubMed Central PMCID: PMCPMC4159683.

14. Lee SM, Kim M, Lee HM, Kwon KY, Koh SB. Nonmotor symptoms in essential tremor: Comparison with Parkinson's disease and normal control. *J Neurol Sci* (2015) 349(1-2):168-73. Epub 2015/02/03. doi: 10.1016/j.jns.2015.01.012. PubMed PMID: 25641389.

15. Lorenz D, Poremba C, Papengut F, Schreiber S, Deuschl G. The psychosocial burden of essential tremor in an outpatient- and a community-based cohort. *Eur J Neurol* (2011) 18(7):972-9. Epub 2011/01/20. doi: 10.1111/j.1468-1331.2010.03295.x. PubMed PMID: 21244579.

16. Louis ED, Benito-León J, Vega-Quiroga S, Bermejo-Pareja F. Faster rate of cognitive decline in essential tremor cases than controls: a prospective study. *Eur J Neurol* (2010) 17(10):1291-7. Epub 2010/06/22. doi: 10.1111/j.1468-1331.2010.03122.x. PubMed PMID: 20561042; PubMed Central PMCID: PMCPMC2939209.

17. Louis ED, Benito-León J, Vega-Quiroga S, Bermejo-Pareja F. Cognitive and motor functional activity in non-demented community-dwelling essential tremor cases. *J Neurol Neurosurg Psychiatry* (2010) 81(9):997-1001. Epub 2010/06/16. doi: 10.1136/jnnp.2009.202838. PubMed PMID: 20547612.

18. Louis ED, Huey ED, Gerbin M, Viner AS. Depressive traits in essential tremor: impact on disability, quality of life, and medication adherence. *Eur J Neurol* (2012) 19(10):1349-54. Epub 2012/05/31. doi: 10.1111/j.1468-1331.2012.03774.x. PubMed PMID: 22642492; PubMed Central PMCID: PMCPMC3434295.

19. Louis ED, Huey ED, Gerbin M, Viner AS. Apathy in essential tremor, dystonia, and Parkinson's disease: a comparison with normal controls. *Mov Disord* (2012) 27(3):432-4. Epub 2011/12/19. doi: 10.1002/mds.24049. PubMed PMID: 22183872.

20. Louis ED, Rao AK, Gerbin M. Functional correlates of gait and balance difficulty in essential tremor: balance confidence, near misses and falls. *Gait Posture* (2012) 35(1):43-7. Epub 2011/09/21. doi: 10.1016/j.gaitpost.2011.08.002. PubMed PMID: 21930384; PubMed Central PMCID: PMCPMC3244510.

21. Louis ED, Viner AS, Gillman A. Mental Status Test Scores are Inversely Correlated with Tremor Severity: A Study of 161 Elderly Essential Tremor Cases. *Tremor Other Hyperkinet Mov (N Y)* (2012) 2. Epub 2013/02/27. doi: 10.7916/d81c1vkg. PubMed PMID: 23440035; PubMed Central PMCID: PMCPMC3409669.

22. Louis ED, Gerbin M, Galecki M. Essential tremor 10, 20, 30, 40: clinical snapshots of the disease by decade of duration. *Eur J Neurol* (2013) 20(6):949-54. Epub 2013/03/26. doi: 10.1111/ene.12123. PubMed PMID: 23521518; PubMed Central PMCID: PMCPMC3653981.

23. Louis ED, Machado DG. Tremor-related quality of life: A comparison of essential tremor vs. Parkinson's disease patients. *Parkinsonism Relat Disord* (2015) 21(7):729-35. Epub 2015/05/09. doi: 10.1016/j.parkreldis.2015.04.019. PubMed PMID: 25952960; PubMed Central PMCID: PMCPMC4764063.

24. Louis ED. More Time with Tremor: The Experience of Essential Tremor Versus Parkinson's Disease Patients. *Mov Disord Clin Pract* (2016) 3(1):36-42. Epub 2016/07/19. doi: 10.1002/mdc3.12207. PubMed PMID: 27430000; PubMed Central PMCID: PMCPMC4943749.

25. Louis ED, Collins K, Rohl B, Morgan S, Robakis D, Huey ED, et al. Self-reported physical activity in essential tremor: Relationship with tremor, balance, and cognitive function. *J Neurol Sci* (2016) 366:240-5. Epub 2016/05/17. doi: 10.1016/j.jns.2016.05.034. PubMed PMID: 27288815.

26. Louis ED, Cosentino S, Huey ED. Depressive symptoms can amplify embarrassment in essential tremor. *J Clin Mov Disord* (2016) 3:11-. doi: 10.1186/s40734-016-0039-6. PubMed PMID: 27429787.

27. Manorenj S, Shravani C, Jawalker S. Clinical Characteristics of Essential Tremor in South India: A Hospital-Based Cohort Study. *J Neurosci Rural Pract* (2019) 10(2):245-9. doi: 10.4103/jnrp.jnrp_348_18. PubMed PMID: 31001012.

28. Monin JK, Gutierrez J, Kellner S, Morgan S, Collins K, Rohl B, et al. Psychological Suffering in Essential Tremor: A Study of Patients and Those Who Are Close to Them. *Tremor Other Hyperkinet Mov (N Y)* (2017) 7:526-. doi: 10.7916/D8Q53WF0. PubMed PMID: 29276649.

29. Morgan S, Kellner S, Gutierrez J, Collins K, Rohl B, Migliore F, et al. The Experience of Essential Tremor Caregivers: Burden and Its Correlates. *Front Neurol* (2017) 8(396). doi: 10.3389/fneur.2017.00396.

30. Musacchio T, Purrer V, Papagianni A, Fleischer A, Mackenrodt D, Malsch C, et al. Non-Motor Symptoms of Essential Tremor Are Independent of Tremor Severity and Have an Impact on Quality of Life. *Tremor Other Hyperkinet Mov (N Y)* (2016) 6:361. Epub 2016/03/19. doi: 10.7916/d8542nch. PubMed PMID: 26989573; PubMed Central PMCID: PMCPMC4790197.

31. Peng J, Wang L, Li N, Li J, Duan L, Peng R. Distinct non-motor features of essential tremor with head tremor patients. *Acta Neurol Scand* (2020) 142(1):74-82. Epub 2020/03/17. doi: 10.1111/ane.13242. PubMed PMID: 32176316.

32. Rao AK, Gillman A, Louis ED. Quantitative gait analysis in essential tremor reveals impairments that are maintained into advanced age. *Gait Posture* (2011) 34(1):65-70. Epub 2011/04/12. doi: 10.1016/j.gaitpost.2011.03.013. PubMed PMID: 21478017; PubMed Central PMCID: PMCPMC3575132.

33. Rao AK, Gilman A, Louis ED. Balance confidence and falls in nondemented essential tremor patients: the role of cognition. *Arch Phys Med Rehabil* (2014) 95(10):1832-7. Epub 2014/04/21. doi: 10.1016/j.apmr.2014.04.001. PubMed PMID: 24769121.

34. Rohl B, Collins K, Morgan S, Cosentino S, Huey ED, Louis ED. Daytime sleepiness and nighttime sleep quality across the full spectrum of cognitive presentations in essential tremor. *J Neurol Sci* (2016) 371:24-31. Epub 2016/10/08. doi: 10.1016/j.jns.2016.10.006. PubMed PMID: 27871441.

35. Sengul Y, Sengul HS, Yucekaya SK, Yucel S, Bakim B, Pazarcı NK, et al. Cognitive functions, fatigue, depression, anxiety, and sleep disturbances: assessment of nonmotor features in young patients with essential tremor. *Acta Neurol Belg* (2015) 115(3):281-7. Epub 2014/12/05. doi: 10.1007/s13760-014-0396-6. PubMed PMID: 25471376.

36. Sengul Y, Sengul HS, Gokcal E, Ustun I, Ozturk A, Yilmaz O, et al. Alexithymia is a non motor symptom of essential tremor regardless of the presence of depression and anxiety. *Neurol Res* (2020) 42(11):946-51. Epub 2020/07/14. doi: 10.1080/01616412.2020.1792702. PubMed PMID: 32657241.

37. Shalash AS, Mohamed H, Mansour AH, Elkady A, Elrassas H, Hamid E, et al. Clinical Profile of Non-Motor Symptoms in Patients with Essential Tremor: Impact on Quality of Life and Age-Related Differences. *Tremor Other Hyperkinet Mov (N Y)* (2019) 9. Epub 2019/12/24. doi: 10.7916/tohm.v0.736. PubMed PMID: 31867132; PubMed Central PMCID: PMCPMC6898893.

38. Smeltere L, Kuzņecovs V, Erts R. Depression and social phobia in essential tremor and Parkinson's disease. *Brain Behav* (2017) 7(9):e00781-e. doi: 10.1002/brb3.781. PubMed PMID: 28948077.

39. Zubair A, Cersonsky TEK, Kellner S, Huey ED, Cosentino S, Louis ED. What Predicts Mortality in Essential Tremor? A Prospective, Longitudinal Study of Elders. *Front Neurol* (2018) 9:1077-. doi: 10.3389/fneur.2018.01077. PubMed PMID: 30581416.
